# Top five unanswered questions in bacterial cell wall research

**DOI:** 10.1016/j.tcsw.2024.100122

**Published:** 2024-02-21

**Authors:** Sarah M. Batt, Katherine A. Abrahams, Gurdyal S. Besra

**Affiliations:** Institute of Microbiology and Infection, School of Biosciences, University of Birmingham, Edgbaston, Birmingham B15 2TT, UK

**Keywords:** Growth phase, Signalling, Regulation

## Introduction

The cell wall and surface of bacteria is crucial to the cell not only to maintain cell shape and osmotic pressure, but also to withstand fluctuating physical and chemical stresses exerted by the environment. It is also an important virulence factor for pathogenic bacteria during attachment and infection, avoiding the immune system and protecting against antibiotics. As such, the bacterial cell wall has been extensively researched, and the fact that so many clinically available antibiotics target cell wall synthesis emphasises its essential role. Here we discuss the five key unanswered questions with respect to the bacterial cell wall.

## Unanswered question #1 – What are the minimum requirements of the cell wall?

Bacteria are categorized into two broad groups based on the structure of the cell wall: Gram-negative (thin peptidoglycan (PG) layer with outer membrane containing lipopolysaccharides (LPS)) and Gram-positive (thick PG layer with teichoic acids). Of course, there are exceptions. ‘Acid-fast’ mycobacteria, have a complex cell wall rich in polysaccharides with an essential terminal mycolic acid layer. While corynebacteria also contain the mycolic acid layer, they can survive without it (Portevin et al., 2004). The LPS layer, though essential for *Escherichia coli* and *Salmonella*, can be deleted in selected strains of *Neisseria, Moraxella* and *Acinetobacter* (Zhang, 2013). Interestingly, some bacterial L-forms and obligate parasite mycoplasmas are cell wall deficient, lacking even the basic PG structure. Pathogenic *Chlamydia* synthesise PG solely at the division plane, leading to speculation that it is a prerequisite for cell division, though this does not account for species lacking PG altogether (Liechti et al., 2016). This leads to a crucial question: which components are essential and why are some bacteria more versatile than others? Within this it is important to understand that essentiality can vary depending on the growth state, along with the extremes of the environmental factors. Virulence is also a factor to consider, and the essentiality of the virulence factors can vary greatly during the infectious cycle. For example, initial macrophage infection by *Mycobacterium tuberculosis* is mediated by surface antigens that bind to an array of host receptors (Schäfer et al., 2009); during latent phase, stimuli such as starvation trigger a change in the lipid profile and thickening of the cell wall that protects from the host’s immune system (Ghazaei, 2018). In Gram-negatives, the expression of surface exposed proteins can change to reflect nutrient requirements or infectious cycles, such as proteins involved in host cell attachment, iron acquisition and motility (van der Woude and Bäumler, 2004). The decoration of the cell wall components can also be highly versatile. In pathogenic bacteria, antigenic variation is a method used to evade the host’s immune system. *Neisseria gonorrhoeae*, for example, vary the subunits of the fimbriae and the sugars of the O-antigen (van der Woude and Bäumler, 2004). Another significant component during infection is the capsule of Gram-positive and Gram-negative bacteria, where both the level of production as well as the sugar components can be varied (van der Woude and Bäumler, 2004). Therefore, in research, it is important to recognise the changing cell wall minimum requirements (dependent on growth/infectious phase and environment), which may impact the direction of research and the ultimate outputs.

## Unanswered question #2 – How is the cell wall remodelled?

We have established that different growth phases, environments or infectious cycles may require the bacteria to adapt the components of the cell wall and we have a comprehensive understanding of its biosynthesis, albeit with gaps in our knowledge. The focus is now diverting to understanding how the cell wall is remodelled and recycled, which, for some bacteria has remained largely understudied. Cell wall remodelling is an essential part of the cell life cycle, wherein up to half the PG can be turned over per generation (Park and Uehara, 2008). Recycling is also particularly important during nutrient starvation and infection. While PG remodelling has been well studied to be a fine balance of lytic enzymes, with a range of bacteria-specific re-uptake and re-utilisation systems, little is known about the remodelling and recycling of the proteins, sugars and lipids of the outer layers of the cell wall. Wall teichoic acids (WTAs) are covalently attached to PG in Gram-positive bacteria, are these recycled with the PG? There is evidence to suggest that the phosphate that they contain is scavenged during limiting conditions (Mayer et al., 2019). We know that PG sugars can be recycled, are there processes for recycling other sugars, for instance during the remodelling of the LPS during infection? In mycobacteria, the trehalose used during mycolic acid transport, is returned to the cytoplasm by LpqY-SugA-SugB-SugC, an ABC transporter essential for virulence (Kalscheuer et al., 2010). Perhaps the multitude of sugar uptake transporters that most bacteria possess double up to recycle cell wall sugars. In Gram-negatives, little is known about outer membrane protein remodelling, though there are clues in the processes that deal with damaged proteins, which involves a set of proteases that are secreted into the periplasm (Rosas and Lithgow, 2022). Are lipids recycled? Undecaprenyl-pyrophosphate (UDP-P/C55-P; DP-P/C50-P in mycobacteria), is a significant lipid carrier utilised by bacteria to transport several components of the cell wall across the inner membrane, including Lipid II in PG synthesis, WTAs, the O-antigen and endobacterial common antigen, along with the sugars arabinose and mannose in mycobacterial species. Drugs or knockouts that affect these synthesis pathways, such as benzothiazinone inhibition of *Corynebacterineae* (Grover et al., 2014) or *lpxC* deletion in *Salmonella* (Zhang et al., 2013), accumulating DP-P/UDP-P-component intermediates, have highlighted the essentiality that the pool of these lipid carriers represents to the bacteria. Therefore, it would be interesting to know how this pool is regulated and recycled; it is likely that the lipid tail sits permanently within the inner membrane while the phosphate head is constantly translocated across with and without its payload. Is the phosphate head flipped back by the same flippase that flips the substrate or is there a specialised transporter, perhaps UppP, the phosphatase that regenerates UDP from UDP-P (Workman and Strynadka, 2020), or the recently discovered UptA and PopT flippases (Roney and Rudner, 2023)? Finally, while asking questions about cell remodelling, it would be interesting to know how the cell coordinates the growth of these intricate layers and how the growth and remodelling is regulated. Within this, how is the thickness of the PG layer controlled? While often thought of as strong and rigid, the PG layer is actually plastic and dynamic, constantly remodelling in response to environmental cues, stress and infection (Cava and Pedro, 2014). Understanding these fundamental processes is essential in the development of future targeted therapeutic interventions.

## Unanswered question #3 – How does the cell wall participate in cell signalling?

The cell wall resides at the epicentre of various signalling processes, many of which lead to a cascade of gene expression that regulates cell wall growth and remodelling, enabling the bacteria to respond to changing environments. The decorated lipid or carbohydrate-moieties integrated or external to the cell wall, secreted autoinducing signals, or cell wall components such as PG fragments liberated during remodelling, play important roles as messenger molecules (Dworkin, 2014). Communications can be considered to be: cell-to-host, cell-to-cell, and to-self. Cell-to-host: with respect to infection, cell wall constituents are recognised by host cells leading to a signalling cascade within the host and triggering components of the innate immune system. The LPS, also known as endotoxin when discussing host interactions, stimulates an inflammatory response and high levels can lead to toxic shock (Sampath, 2018). Extracellular vesicles bud out from the outer membrane carrying cargoes of LPS, PG fragments, proteins or nucleic acid, that can either enhance symbiotic relationships for gut microbiota, or deliver virulence factors in pathogenesis (Schwechheimer and Kuehn, 2015). Cell-to-cell: quorum sensing is a well-known signalling process that detects and responds to fluctuating population densities via the concentrations of secreted autoinducer molecules. Other signals, such as the release of muropeptides from neighbouring cells, can stimulate growth resumption from dormancy and germination from spores (Jõers et al., 2019, Shah et al., 2008). Interestingly, stimulation of growth by muropeptides in Gram-positive and Gram-negative bacteria occurs through different pathways, demonstrating convergent evolution and exemplifying the importance of muropeptide release and detection to communicate microbial growth and optimal environment (Jõers et al., 2019). Given the relatively new field of wall remodelling and recycling, many details, such as the muropeptide receptors, are undetermined and the current knowledge provides a platform for this area to be further investigated. In addition to roles in host interactions, extracellular vesicles are important for both inter- and intra-bacterial species communications, regulating biofilm formation in response to stress or delivering enzymes for nutrient acquisition in the host’s gut (Schwechheimer and Kuehn, 2015). To-self: Sampling and internal communication of growth phase and external environments (such as nutrient availability, pH, temperature, antimicrobials to name but a few), allows the bacteria to adjust the composition of the cell wall by the spatial and temporal regulation of substrates and metabolic machinery. For example, the fluidity of the membrane is regulated by altering the chain length of the membrane lipids to adapt to different temperatures, or the porins of the outer membrane can be varied to prevent antibiotic uptake (Rosas and Lithgow, 2022). The response to external stimuli is controlled by a two-component regulatory system, which alters the gene expression profile (Hirakawa et al., 2020). The existence of cell wall components is another method of internal communication. For example, in *Staphylococcus aureus*, WTAs temporally and spatially control the level of PG cross-linking (Atilano et al., 2010) and the removal of both lipo- and WTAs prevent FtsZ ring assembly during division (Santa Maria et al., 2014). The relative levels of muropeptides can also be used to sense the presence of β-lactam antibiotics, signalling to upregulate resistance genes (Jacobs, et al., 1997). Further research is required to unravel the details in these complex and diverse signalling pathways.

## Unanswered question #4 – How dynamic is the cell wall?

A fundamental feature of the cell wall is its dynamic properties. It maintains cell integrity in changing physical and chemical environments, adapting its permeability barrier and nutrient uptake, modulating virulence and antibiotic susceptibility and controlling cell signalling whilst also regulating growth and division. Cell wall mechanics are directly controlled by the several components: Gram-negative LPS impacts the permeability, the length and cross-linking of PG chains affects the stiffness of the sacculus, and the constituents of the lipid layers effects rigidity, which can vary with temperature. Although we can categorically answer the above question, the cell wall is a very dynamic structure, the intricate mechanisms involved in regulating and distributing biochemical machinery and lipid composition are not entirely understood. Studies on the dynamics of the cell wall are gaining momentum following the application of new imaging techniques to visualise cell wall architecture. Cryo-electron tomography and fluorescence microscopy have propelled the understanding of the cell wall dynamics, giving new insights into the different stages of cell growth and division (Navarro et al., 2022). The ability to depict the individual layers of the cell wall has enabled the impact and the roles of enzymes involved in cell wall metabolism to be further investigated, either through mutations, gene knockout or inhibitor studies. Fluorescence imaging techniques such as fluorescence recovery after photobleaching (FRAP) have also been incremental in studying cell wall dynamics in live cells. Fluorescent analogues of cell wall components have been used as probes, incorporating into the cell wall, enabling the quantification, subcellular organisation and diffusion dynamics of membrane constituents. This technique has been used to show the impact of inhibitors of cell wall biosynthesis. For example, treatment of *Corynebacterium* with ethambutol, an antibiotic that inhibits an enzyme in arabinan biosynthesis, causes a loss of mycolic acid attachment sites and a mislocalisation of apical growth machinery visualised by markers of PG biosynthesis (Rodriguez-Rivera et al., 2017, Schubert et al., 2017). This highlights the dynamic interplay between different cell wall layers, and the importance of timely localization of metabolic machinery and their substrates. Advances in computer simulations have also provided a powerful tool, computing experimentally obtained parameters to predict dynamics, such as confirming the predicted asymmetry of the plasma membrane glycolipids of mycobacteria (Brown,et al., 2023). The ability to now temporally and spatially resolve cell wall components and biosynthetic machinery should further enhance our understanding of the complex cell wall dynamics and reveal the answer to one of the many questions: do the fundamental differences in cell wall architecture between bacteria arise from variances in the biosynthetic pathways, or from differences in the spatial and temporal regulation of the substrates and machinery?

## Unanswered question #5 – What is the function of the putative proteins?

While many cell wall synthesis pathways have been studied and functions assigned, there are still many genes even for the most prominent bacteria that encode uncharacterised proteins with no known function. Recent advances in artificial intelligence (AI) are already beginning to address this issue. AlphaFold, for example, has revolutionised the field of structural biology, providing a database of accurately predicted protein structures that can be used to infer functions and interactions based on folds and active site similarities. Along with this, the facility to model protein interactions in multimeric structures will be instrumental in predicting synthesis pathways and the assembly of large protein ‘factories’ (Jumper et al., 2021). Future AI could be used to amalgamate databases, streamlining gene names and citing literature, and assigning putative functions by comparing protein sequences and predicted structure/functions of a hypothetical protein across all of the ‘knowns’ from all bacterial species. This would certainly be advantageous in unravelling of the roles of putative proteins in organisms of interest and propel research efforts. In addition, it would be interesting to know why are there so many apparently redundant non-essential genes? Genome size can vary greatly depending on environmental pressures and one third of the bacterial chromosome usually encodes membrane proteins involved in membrane synthesis and substrate transport, again emphasising the significance of the cell wall (Jeckelmann and Erni, 2020). Bacteria that need to adjust to rapidly changing environments may have several transporters for the same substrate, for example, *E. coli* has seven transporters with overlapping substrates such as glucose (Jeckelmann and Erni, 2020). Redundancy has also been observed for the fatty acid β-oxidation pathways of *M. tuberculosis* and is speculated to enable the bacterium to switch metabolism depending on available nutrients, the EchA genes for example are differentially expressed during nutrient starvation (Williams et al., 2011). The fact that these apparently redundant genes persist in bacterial populations does imply there could be evolutionary pressures at work here. Therefore ‘redundancy’ in genomics may just be the ability to adjust to fluctuating environments and ‘essentiality’ depends on the conditions and growth/infectious cycle. Thus, versatility is likely to be related to genome size and the relative abundance of ‘redundant’ genes.

## Concluding remarks

The unanswered questions discussed above cover broad areas of research and are by no means exhaustive. Whilst aspects of each question can be answered thanks to significant progress in the development of new techniques and technologies forged over recent years, there are still many unknowns. We hope that this article prompts new ideas and inspires future scientists to answer these valuable queries and generate new ones in an ever-evolving complex field of bacteriology.

## Competing interest statement

The authors declare no competing interest.

## Funding

Personal Research Chair from Mr. James Bardrick (GSB).

Medical Research Council MR/S000542/1.

Medical Research Council MR/R001154/1.

## CRediT authorship contribution statement

**Sarah M. Batt:** Writing – review & editing, Writing – original draft, Visualization, Conceptualization. **Katherine A. Abrahams:** Writing – review & editing, Writing – original draft, Visualization. **Gurdyal S. Besra:** Writing – review & editing, Writing – original draft, Visualization, Supervision, Funding acquisition, Conceptualization.

## Declaration of competing interest

The authors declare the following financial interests/personal relationships which may be considered as potential competing interests: Gurdyal Singh Besra reports financial support was provided by Medical Research Council and a relationship with Microbiology Society that includes: travel reimbursement.
